# EPIGENETIC AGING AND RHEUMATOID ARTHRITIS

**DOI:** 10.1093/geroni/igad104.2486

**Published:** 2023-12-21

**Authors:** Nandini Mukherjee, Tracie Harrison

**Affiliations:** The University of Arkansas for Medical Sciences, Little Rock, Arkansas, United States; The University of Arkansas for Medical Sciences, Little Rock, Arkansas, United States

## Abstract

Indicators of biological aging with rheumatoid arthritis (RA), a chronic autoimmune disease, are not yet fully known. In this cross-sectional study, we tested associations between plasma indicators of epigenetic aging using online data (GSE42861) regarding RA and DNA methylation from the Swedish Epidemiological Investigation of Rheumatoid Arthritis (EIRA). Peripheral blood DNA methylation of participants 18-70 years old was assessed using Illumina HumanMethylation450 arrays. Horvath’s online DNA Methylation Age Calculator determined DNAm estimate of Telomere length (DNAmTL), Hannum’s, Horvath’s 2013 and 2018 epigenetic ages, PhenoAge and GrimAge and the respective age accelerations. Association of RA prevalence with epigenetic age were determined using linear regression, correcting for false discovery rate. We identified significant associations of RA with age acceleration measures of Horvath 2013 (Estimate:-1.34 ; FDR p-value: 1.0 × 10-2) and Horvath 2018 (Estimate:-1.32; FDR p-value: 4.0 × 10-5), extrinsic age acceleration (Estimate: 1.34 ; FDR p-value: 1.0 × 10-2), PhenoAge acceleration (Estimate: 2.31; FDR p-value: 1.1 × 10-5), GrimAge (Estimate: 2.54; FDR p-value: 1.0 × 10-2) and GrimAge acceleration (Estimate: 3.15 ; FDR p-value: 1.7 × 10-17), adjusting for sex and smoking (FDR p-value<=0.05). In sex stratified analyses, first-generation clocks were associated only in women, but no sex specific effects for PhenoAge or GrimAge accelerations were identified. The second generation ‘biological’ clocks PhenoAge, GrimAge and its components showed higher epigenetic age acceleration in RA cases compared to healthy controls. We conclude that second generation indicators of epigenetic aging provide promise as markers of biological aging in people with RA.

